# Specific *HER2* Exon 20 Gly776 Deletion-Insertions in Non-Small Cell Lung Cancer: Structural Analysis and Sensitivity to *HER2*-Targeted Tyrosine Kinase Inhibitors

**DOI:** 10.3389/fphar.2022.806737

**Published:** 2022-03-07

**Authors:** Guangjian Yang, Haiyan Xu, Jiaqi Hu, Runze Liu, Peizeng Hu, Yaning Yang, Weihua Li, Xuezhi Hao, Shuyang Zhang, Fei Xu, Xin Ai, Junling Li, Yan Wang

**Affiliations:** ^1^ Department of Medical Oncology, National Cancer Center/National Clinical Research Center for Cancer/Cancer Hospital, Chinese Academy of Medical Sciences and Peking Union Medical College, Beijing, China; ^2^ Department of Comprehensive Oncology, National Cancer Center/National Clinical Research Center for Cancer/Cancer Hospital, Chinese Academy of Medical Sciences and Peking Union Medical College, Beijing, China; ^3^ Drug Discovery Business Unit, PharmaBlock Sciences (Nanjing), Inc., Nanjing, China; ^4^ Graduate School, Guangxi Medical University, Nanning, China; ^5^ Department of Traditional Chinese Medicine, Yidu Central Hospital of Weifang, Qingzhou, China; ^6^ Department of Pathology, National Cancer Center/National Clinical Research Center for Cancer/Cancer Hospital, Chinese Academy of Medical Sciences and Peking Union Medical College, Beijing, China

**Keywords:** *HER2* exon 20 insertion, afatinib, tyrosine kinase inhibitor, Gly776 deletion-insertion, non-small cell lung cancer, pyrotinib

## Abstract

**Background:** *HER2* exon 20 insertions remain a subset heterogeneous alterations in lung cancer, with currently unmet need for precision targeted therapy. G776delinsVC, a typical *HER2* exon 20 deletion-insertion at codon Gly776, was reported to respond discrepantly to afatinib compared with the predominant insertion A775_G776insYVMA (YVMA). However, it lacks structural evidence to illustrate the possible mechanism and predict the binding activities of its similar variants over YVMA insertion to *HER2*-targered tyrosine kinase inhibitors (TKIs).

**Methods:** Real-world cohort study was performed to investigate clinical outcomes with *HER2*-targeted TKI afatinib and pyrotinib, and structural analysis for exon 20 Gly776 deletion-insertions G776delinsVC, G776delinsLC and G776delinsVV, and YVMA by molecular dynamics simulation and cellular kinase inhibition assay were provided for full exploration.

**Results:** Afatinib revealed low objective response rate (ORR) of 0–9.5% and short median progression-free survival (mPFS) of 2.8–3.2 months for YVMA, but with higher ORR of 20–28.6% and longer mPFS of 4.3–7.1 months for G776delinsVC. Pyrotinib presented significantly improved PFS benefit than afatinib for G776delinsVC and YVMA as first-line (median, 6.8 *vs.* 3.4 months, *p* = 0.010) or second-line therapy (median, 5.8 *vs.* 2.8 months, *p* < 0.001). No significant difference was observed on drug binding pocket and TKI binding activity between G776delinsVC, G776delinsLC and G776delinsVV, and both afatinib and pyrotinib showed favorable binding activity. YVMA insertion significantly affected the loop region with altering *HER2* protein secondary structure and forming steric hindrance to binding of afatinib. Pyrotinib showed the best selectivity to *HER2*, with more favorable activity to YVMA than afatinib indicated by cellular inhibition assay.

**Conclusion:** Both afatinib and pyrotinib showed favorable activity for NSCLC patients with *HER2* exon 20 Gly776 deletion-insertions. Pyrotinib revealed more potent activity to A775_G776insYVMA insertion than afatinib due to the steric binding hindrance induced by YVMA.

## Introduction

Activating alterations of human epidermal growth factor receptor 2 (*HER2*, also known as *ERBB2*), a member of the *ErbB* receptor tyrosine kinase family, have been identified as oncogenic drivers, occurring in 2–4% in non-small cell lung cancer (NSCLC) (Stephens et al., 2004; Arcila et al., 2012; Mazières et al., 2013; Peters and Zimmermann, 2014; Kris et al., 2015). The 12-base pair (bp) in-frame insertion A775_G776insYVMA (YVMA), resulting in duplication of four amino acids Tyrosine-Valine-Methionine-Alanine (Tyr-Val-Met-Ala) in exon 20, is observed the most frequent *HER2* alteration variant in NSCLC, which is accounting for approximately 60–70% of *HER2* exon 20 insertions (ex20ins) (Shigematsu et al., 2005; Perera et al., 2009; Arcila et al., 2012; Liu et al., 2018; Jebbink et al., 2020). With functional significance, ex20ins lead to constitutive activation of *HER2* tyrosine kinase activity and initiate the protumorigenic downstream *MEK*/*ERK*/*MAPK* and *PI3K*/*AKT*/*mTOR* pathway signaling cascade, resulting in increased proliferation and tumor growth (Berns et al., 2007; Spector and Blackwell, 2009; Arcila et al., 2012; Hanker et al., 2013).

To date, several studies had reported response heterogeneities to *HER2*-targeted tyrosine kinase inhibitor (TKI) afatinib among *HER2* ex20ins (De Grève et al., 2012; Liu et al., 2018; Nagano et al., 2018; Dziadziuszko et al., 2019; Lai et al., 2019). The prospective NICHE study terminated early with the conclusion that afatinib failed to show the expected benefit for *HER2* ex20ins patients, especially for those with YVMA insertion, with a short median progression-free survival (mPFS) of 15.9 weeks and poor objective response rate (ORR) of 7.7% (Dziadziuszko et al., 2019). Similarly, a multicenter retrospective study from China as well issued afatinib just achieved a short mPFS of 3.7 months and ORR of 18.2% (2/11) for YVMA insertion (Liu et al., 2018). In addition, afatinib was reported with significantly improved mPFS for non-YVMA insertions (G778_P780dup and G776delinsVC) compared with YVMA (7.6 *vs.* 1.2 months, *p* < 0.001), in which no response (ORR, 0/14) was observed in patients with YVMA (Kosaka et al., 2017; Fang et al., 2020). In summary, these studies revealed that YVMA insertion showed relatively much lower response to afatinib than other *HER2* ex20ins variants. As a novel pan-*ErbB* inhibitor, pyrotinib potently inhibits *HER2* and has been reported with promising and broader efficacy than afatinib for various *HER2* ex20ins variants in both patient-derived xenograft (PDX) model and cohort studies (Wang et al., 2019; Zhou et al., 2020; Yang et al., 2021).

Three-dimensional (3D) modeling has demonstrated that *HER2* ex20ins induced a constitutively active conformation and caused steric hindrance of the drug-binding pocket, preventing the binding of the noncovalent *HER2*-targeted TKIs (Robichaux et al., 2018). As a clinically active inhibitor, poziotinib binds tightly into the sterically hindered drug-binding pocket of YVMA insertion to overcome structural changes and inhibit *HER2*-mutant NSCLC more potently than afatinib by *in vitro* evidence (Robichaux et al., 2018). Furthermore, the ZENITH20-2 study of poziotinib showed that the mPFS was 5.5 months and the ORR was 27% in advanced NSCLC patients with *HER2* ex20ins (Elamin et al., 2021). In addition, the hypoxia-activated prodrug tarloxotinib, concentrating high levels of active drug tarloxotinib-E (a potent and covalent pan-*ErbB* inhibitor) within the tumor microenvironment than normal tissue, has been reported of marked tumor response in *HER2*-activating mutations in NSCLC observed in the RAIN-701 study and *in vitro* models (Liu et al., 2020; Estrada-Bernal et al., 2021).

G776delinsVC, another specific ex20ins subtype composed of insertion of amino acids Valine and Cysteine (Val-Cys, VC) instead of original glycine at codon 776 (Gly776), was reported to derive more benefit from afatinib when compared with YVMA insertion, but the sample size was too small, with only 5 cases (Fang et al., 2020). In addition, another study found G776delinsVC displayed very different responses to afatinib (Liu et al., 2018). However, currently, it lacks structural research distinguishing the molecular heterogeneities between G776delinsVC and its similar variants G776delinsVV and G776delinsLC, especially lacking evidence to predict drug sensitivities to afatinib and pyrotinib for these specific Gly776 deletion-insertion subtypes. It is warranted to give a deeper investigation into their molecular structure to guide precision targeted therapy with anti-*HER2* TKIs. In this study, we investigated responses to afatinib and pyrotinib for *HER2* Gly776 deletion-insertions from a real-world cohort study, and concomitantly explored to identify their molecular differences over YVMA insertion by structural analysis and cellular kinase inhibition assay.

## Materials and Methods

### Patients and Molecular Detection

We retrospectively reviewed 172 patients with histologically confirmed metastatic or recurrent NSCLC harboring certain *HER2* ex20ins subtypes of A775_G776insYVMA (c.2324_2325insCACCGTGATGGC), G776delinsVC (c.2326_2327insTGT), G776delinsLC (c.2326delinsTTGT) and G776delinsVV (c. 2327delinsTTGT), who had received at least one systemic therapy regimen from January 2016 to June 2021 recorded by medical database in Chinese Academy of Medical Sciences (CAMS)/Cancer Hospital. Information regarding clinical characteristics, *HER2* ex20ins variants, and treatment outcome with afatinib or pyrotinib from medical records were collected. *HER2* ex20ins were identified using next-generation sequencing (NGS) based on specimens of tumor tissue or plasma. Molecular testing data of *HER2* ex20ins among these patients were both collected from the department of pathology in CAMS/Cancer Hospital, and from patients who performed *HER2* ex20ins testing by qualified third-party genetic testing companies which had passed the national quality system certification in China. All of the NGS testing for *HER2* ex20ins was performed based on the Illumina sequencing system. This retrospective study was approved by the ethical committee of National Cancer Center, and written informed consent was waived.

### Efficacy Evaluation

Tumor response was evaluated using computed tomography images of chest and abdomen and brain magnetic resonance imaging, according to the Response Evaluation Criteria in Solid Tumors (RECIST) version 1.1. PFS was defined as time from initiation of *HER2*-targeted TKIs to date of documented disease progression or death from any cause (whichever occurs first). ORR was defined as the proportion of patients with at least one confirmed complete response (CR) and partial response (PR) after TKI targeted therapy. Disease control rate (DCR) was defined as the proportion of patients with at least one confirmed CR, PR and stable disease (SD) by *HER2*-targeted TKIs.

### Computational Structure and Molecular Dynamics Simulation

The homology models of four *HER2* ex20ins variants (A775_G776insYVMA, G776delinsVC, G776delinsLC, G776delinsVV) were computationally constructed based on the crystal structure of human *HER2* kinase domain in complex with inhibitor TAK285 by the Schrödinger software (2021-1 Release, Schrödinger Inc., Portland, Oregon) (PDB ID: 3PP0). The protein was prepared using Maestro software (Schrödinger 2021-1 Release) in the Schrödinger modeling package. Compounds were constructed using the 3D-sketcher module in Maestro. Binding free energy (ΔG_*bind*_) was used to evaluate the binding activity of a certain compound, which was calculated by molecular mechanics/Poisson-Boltzmann solvent accessible surface area (MM/PBSA) and molecular mechanics/Generalized Born solvent accessible surface area (MM/GBSA) methods. The detailed calculation regarding ΔG_*bind*_ of a protein-ligand complex was listed in the Appendix.

### Cell Lines and Kinase Inhibition Assay

In order to evaluate the inhibitory ability of compounds synthesized on the proliferation of A775_G776insYVMA and *HER2* wild type (WT) Ba/F3 cells (purchased from Beijing Kangyuan Bochuang Biotechnology Co., Ltd.), exponentially growing cells were seeded in the media of RPMI Medium 1640 basic with 1% penicillin-streptomycin (purchased from ThermoFisher, Waltham, MA, United States) and 10% Foetal Bovine Serum (FBS, purchased from Biological Industries) at a concentration of 1000 cells/ml in a 384-well cell culture plate (purchased from CORNING, United States) with 20 µl per well and incubated overnight at 37°C, 5% CO_2_ incubator. Compounds were prepared as 12-point, 3-fold serial dilutions in dimethyl sulfoxide (DMSO), beginning at 2 mM. 1 µl of DMSO solutions from the compound stock plates were added to 99 µl of cell culture media (final top concentration of compound in the assay was 10 µM and the final concentration of DMSO was 0.5%). 20 µl of compound solutions in media were added to Ba/F3 cell plates. After adding compound solutions, assay plates were incubated for 3 days at 37°C, 5% CO_2_. Cell viability was measured using the CellTiter-Glo assay kit from Promega (Madison, WI, United States) by quantitating the adenosine triphosphate (ATP) present in the cell cultures. Luminescence was read after 20 min of incubation with the SPARK multiple plate reader from TECAN (Switzerland). The half maximal inhibitory concentrations (IC_50_) of compounds inhibiting cell viability were determined using a sigmoidal dose-response model (variable slopes, four parameters) in Prism 7 (La Jolla, CA) to evaluate the inhibitory ability of compounds on the proliferation of *HER2*-WT and YVMA Ba/F3 cells. Each experiment was replicated three separate times to give biological replicates unless indicated otherwise.

### Statistical Analysis

The Chi-Square test and Fisher’s exact test were applied to compare the significance of baseline differences between subgroups. Statistical analyses were conducted using SAS^®^ software (version 9.4, SAS Institute Inc, Cary, United States). PFS were analyzed using the Kaplan-Meier method. PFS between different subgroups were compared using the log-rank test (two-sided), and corresponding 95% confidence interval (CI) were estimated using Cox proportional regression model. *p* value < 0.05 was considered statistically significant.

## Results

### Overview of Patients’ Characteristics

A total of 172 eligible patients with metastatic or recurrent lung adenocarcinoma who received chemotherapy, immune checkpoint inhibitors or TKIs were screened in this study. Among the entire population, 42 patients received first-line (1L) targeted therapies including *HER2*-targeted TKI afatinib (n = 26), pyrotinib (n = 10) and poziotinib (n = 1), T-DM1 (n = 1), first-generation *EGFR* TKIs (n = 3) and antiangiogenic TKI anlotinib (n = 1). For second-line (2L) targeted therapy, 55 cases administered *HER2*-targeted TKIs, including afatinib (n = 24) and pyrotinib (n = 31). The last follow-up date was 26 June 2021. Patients’ characteristics with 1L and 2L *HER2*-targeted TKIs are summarized in Table 1.

**TABLE 1:** Clinicopathological characteristics of patients with *HER2* ex20ins by 1L and 2L targeted therapies.

Characteristics	1L total	Afatinib	Pyrotinib	*p*-Value	2L total	Afatinib	Pyrotinib	χ^2^	*p*-Value
N (%)	36	26	10		55	24	31		
**Age**									
≤55	10 (27.8)	6 (23.1)	4 (40.0)	0.41	30 (54.5)	14 (58.3)	16 (51.6)	0.25	0.62
>55	26 (72.2)	20 (76.9)	6 (60.0)		25 (45.5)	10 (41.7)	15 (48.4)		
**Gender**									
Female	20 (55.6)	14 (53.8)	6 (60.0)	1.0	32 (58.2)	15 (62.5)	17 (54.8)	0.33	0.57
Male	16 (44.4)	12 (46.2)	4 (40.0)		23 (41.8)	9 (37.5)	14 (45.2)		
**Smoking history**									
Never	24 (66.7)	17 (65.4)	7 (70.0)	1.0	40 (72.7)	21 (87.5)	19 (61.3)	4.69	0.03
Current/Former	12 (33.3)	9 (34.6)	3 (30.0)		15 (27.3)	3 (12.5)	12 (38.7)		
***HER2* testing specimen**									
Tumor tissue	34 (94.4)	24 (92.3)	10 (100.0)	1.0	52 (94.5)	22 (91.7)	30 (96.8)	-	0.58
Plasma	2 (5.6)	2 (7.7)	0 (0)		3 (5.5)	2 (8.3)	1 (3.2)		
***HER2* ex20ins variants**									
A775_G776insYVMA	29 (80.6)	21 (80.8)	8 (80.0)	1.0	45 (81.8)	17 (70.9)	28 (90.3)	-	0.08
G776delinsVC	7 (19.4)	5 (19.2)	2 (20.0)		6 (11.0)	3 (12.5)	3 (9.7)		
G776delinsVV	0 (0)	0 (0)	0 (0)		2 (3.6)	2 (8.3)	0 (0)		
G776delinsLC	0 (0)	0 (0)	0 (0)		2 (3.6)	2 (8.3)	0 (0)		
**Brain metastasis**									
Presence	7 (19.4)	5 (19.2)	2 (20.0)	1.0	5 (9.1)	2 (8.3)	3 (9.7)	0.003	0.96
Absence	29 (80.6)	21 (80.8)	8 (80.0)		50 (90.9)	22 (91.7)	28 (90.3)		

### Clinical Efficacy of *HER2*-Targeted TKIs

#### Afatinib

**FIGURE 1:**
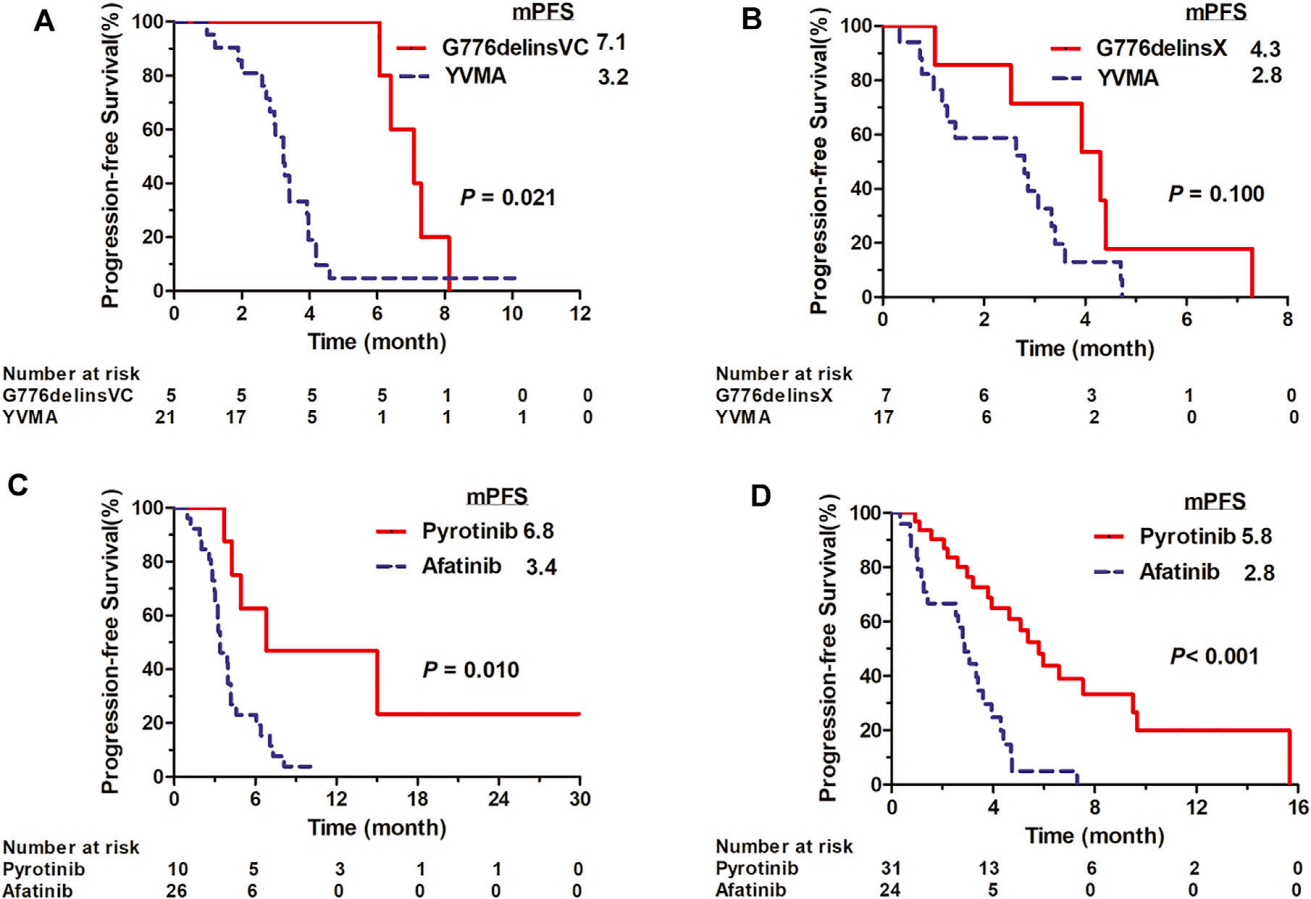
Kaplan-Meier curves for PFS of afatinib for distinct *HER2* ex20ins variants G776delinsVC and A775_G776insYVMA in first-line **(A)** and second-line setting **(B)**. Kaplan-Meier curves for PFS of pyrotinib *vs.* afatinib among overall ex20ins patients as first-line **(C)** and second-line therapy **(D)**.

In 1L setting, afatinib showed mPFS of 3.4 months (95% CI: 2.528–4.272) in total 26 cases, with an overall ORR of 11.5% (3/26) and DCR of 80.8% (21/26). It revealed ORR of 9.5% (2/21), DCR of 81.0% (17/21) and mPFS of 3.2 months (95% CI: 2.826–3.634) in YVMA subgroup (n = 21), and ORR of 20% (1/5), DCR of 80% (4/5) and significantly improved mPFS of 7.1 months (95% CI: 5.597–8.603) for patients harboring G776delinsVC (*p* = 0.021) (Figure 1A). In 2L setting, it revealed overall ORR of 8.3% (2/24), DCR of 66.7% (16/24) and mPFS of 2.9 months (95% CI: 2.199–3.541) among total 24 cases. For 17 patients with YVMA insertion, it showed no response, DCR of 58.8% (10/17) and mPFS of 2.8 months (95% CI: 1.022–4.578), whereas, it revealed ORR of 28.6% (2/7), DCR of 85.7% (6/7) and mPFS of 4.3 months (95% CI: 2.377–6.223) for patients with G776delinsX (G776delinsVC, G776delinsLC and G776delinsVV) (*p* = 0.100) (Figure 1B).

#### Pyrotinib

Pyrotinib showed mPFS of 6.8 months (95% CI: 0–16.652) in total 10 cases as 1L therapy, with an overall ORR of 22.2% (2/9, one case not evaluable) and DCR of 100% (9/9). For YVMA insertion (n = 8), it revealed ORR of 14.2% (1/7, one case not evaluable), DCR of 100% (7/7) and mPFS of 4.9 months (95% CI: 1.893–7.967). Among two patients with G776delinsVC, one showed PR to pyrotinib with PFS of 15.0 months, and the other one revealed an ongoing PFS of 5.5 months with SD as the best response. Generally, pyrotinib revealed significant PFS benefit compared with afatinib in overall patients as 1L therapy (median, 6.8 *vs.* 3.4 months, *p* < 0.010) (Figure 1C). Pyrotinib showed mPFS of 5.8 months (95% CI: 4.419–7.181) with an ORR of 14.8% (4/27, one case not evaluable) and DCR of 92.6% (25/27) for YVMA insertion (n = 28) in 2L setting. Among three patients with G776delinsVC, one showed PFS of 3.8 months with the best response of SD, and the other 2 cases achieved ongoing PFS of 5.3 months with PR, and ongoing PFS of 4.6 months with SD as best response until to the last follow-up time. Totally, 2L pyrotinib therapy revealed significantly improved PFS benefit than afatinib among overall patients (median, 5.8 *vs.* 2.8 months, *p* < 0.001) (Figure 1D).

### 3D Modelling and TKI Binding Simulation

**FIGURE 2:**
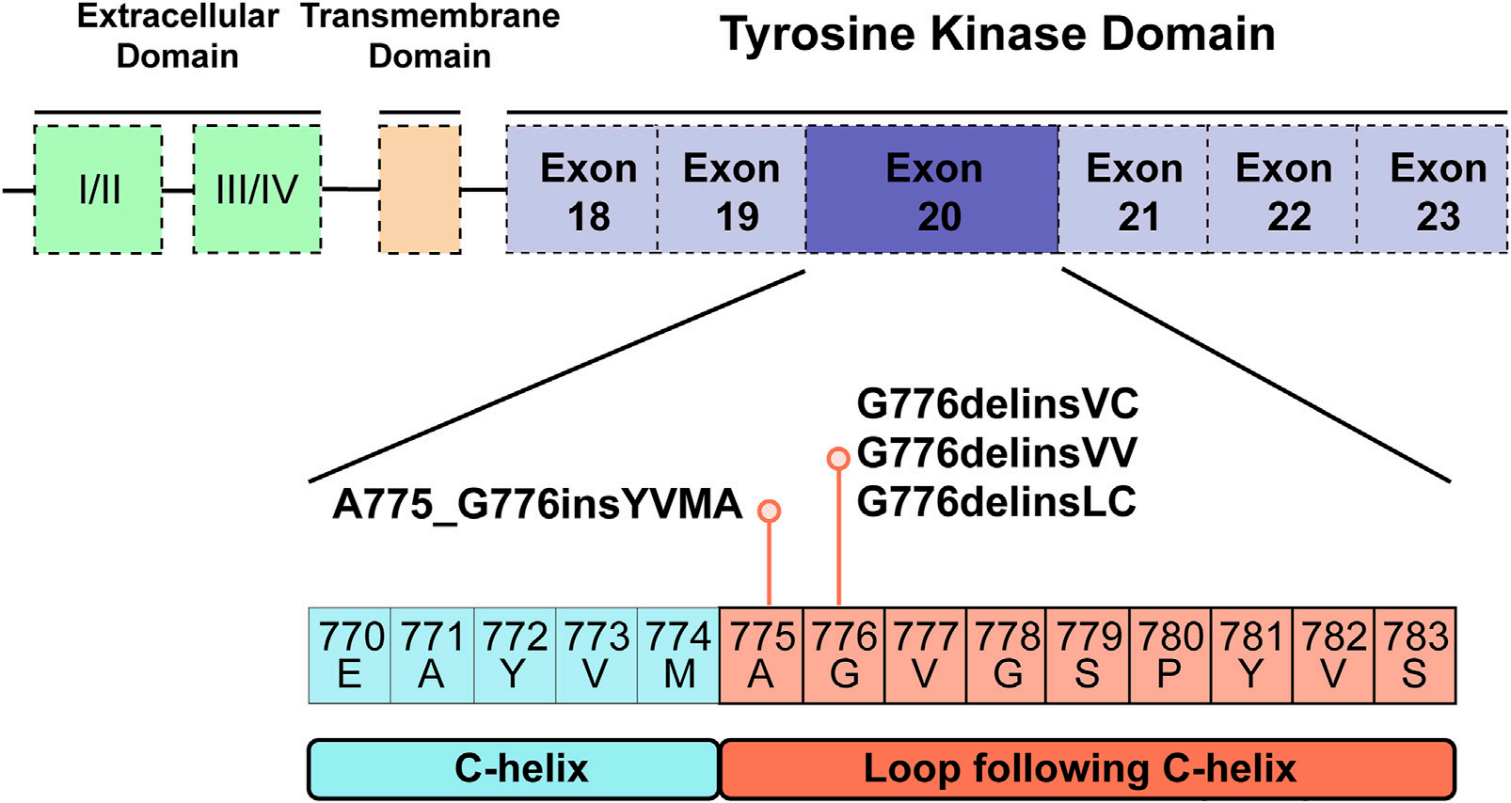
Tyrosine kinase domain of *HER2* and specific exon 20 Gly776 deletion-insertions G776delinsVC, G776delinsLC and G776delinsVV, and A775_G776insYVMA located in the ATP-binding loop region following the C-helix.

**FIGURE 3:**
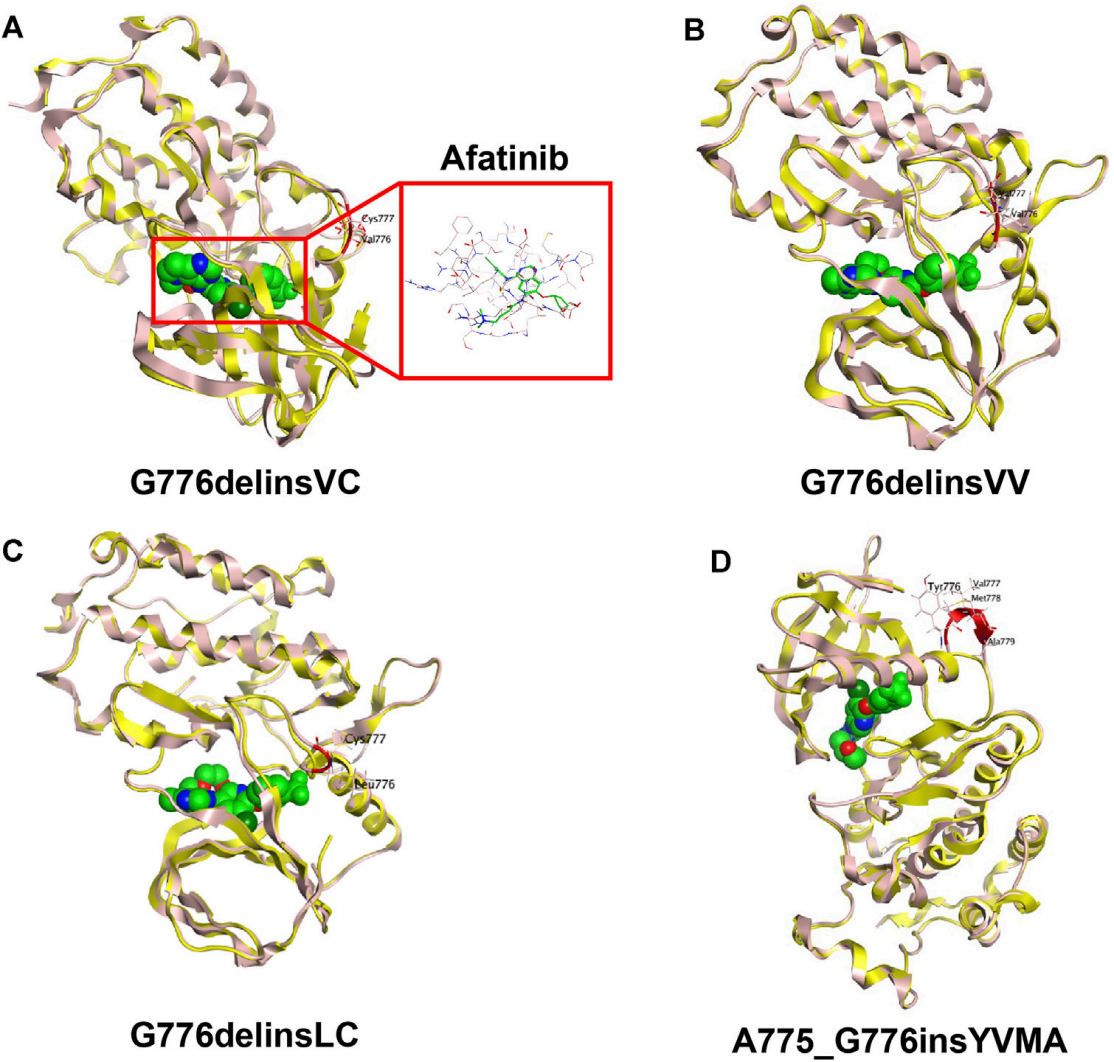
3D-based homology protein models (*HER2* wild type marked in yellow, *HER2* mutants marked in pink) of *HER2* exon 20 Gly776 deletion-insertion variant G776delinsVC with the small molecule TKI afatinib **(A)**, G776delinsVV **(B)**, G776delinsLC **(C)**, compared with the most common 12-bp in-frame insertion A775_G776insYVMA **(D)**.

For human *HER2* kinase domain, the exon 20 Gly776 deletion-insertions (G776delinsVC, G776delinsVV and G776delinsLC) and 12-bp insertion A775_G776insYVMA are all located in the ATP-binding kinase domain, especially in the loop region following the C-helix (Figure 2). For structural analysis of G776delinsVC, the deletion-insertion position is in the loop region from residue Ala775 to Arg784, which is flexible in the kinase domain. The two inserted amino acid residues Val776 and Cys777 just slightly affect the loop region, neither with effect on the overall conformation of *HER2* kinase domain nor the binding pocket, which less likely affects the binding activity to afatinib (Figure 3A). Similar results are observed when amino acids Val776 and Val777 insert instead of the original residue Gly776 (G776delinsVV, Figure 3B), or inserting amino acids Leu776 and Cys777 (G776delinsLC, Figure 3C), indicating Gly776 deletion-insertions seldom shifting the drug binding pocket and overall conformation of *HER2* kinase domain, thus exerting little influence on binding affinity to afatinib. As was illustrated in Table 2, afatinib revealed good binding activity to these three Gly776 deletion-insertion variants, and pyrotinib revealed lower ΔG_*bind*_ values compared to afatinib, indicating a more potent binding activity than afatinib. Of note, for the typical Gly776 deletion-insertion subtype G776delinsVC, four kinds of *HER2*-targeted TKIs (pyrotinib, afatinib, dacomitinib, and poziotinib) all exhibited favorable ΔG_*bind*_ values, in which pyrotinib showed the most potent binding activity (Table 3).

**TABLE 2:** Binding energy of *HER2*-targeted inhibitor afatinib and pyrotinib for Gly776 deletion-insertion variants.

Binding energy (kcal/mol)	G776delinsVC		G776delinsLC		G776delinsVV	
	Pyrotinib	Afatinib	Pyrotinib	Afatinib	Pyrotinib	Afatinib
ΔE_*ele*_	−24.64	−25.17	−38.73	−17.78	−24.93	−17.77
ΔE_*vdW*_	−69.40	−57.09	−74.42	−50.74	−71.03	−56.70
ΔG_*bind*_ (MM/PBSA)	−55.23	−48.50	−43.22	−42.63	−55.82	−44.88
ΔG_*bind*_ (MM/GBSA)	−55.94	−50.06	−59.31	−44.00	−57.83	−46.70

**TABLE 3:** Binding energy of *HER2*-targeted TKIs towards *HER2* ex20ins variant G776delinsVC.

Molecule	Δ*E*_*ele*_	Δ*E*_*vdw*_	ΔG_*bind*_ (MM/PBSA)	ΔG_*bind*_ (MM/GBSA)
Pyrotinib	−24.64	−69.40	−55.23	−55.94
Afatinib	−25.17	−57.09	−48.50	−50.06
Dacomitinib	−11.66	−58.32	−47.40	−51.94
Poziotinib	−11.80	−53.53	−44.44	−45.36

**FIGURE 4:**
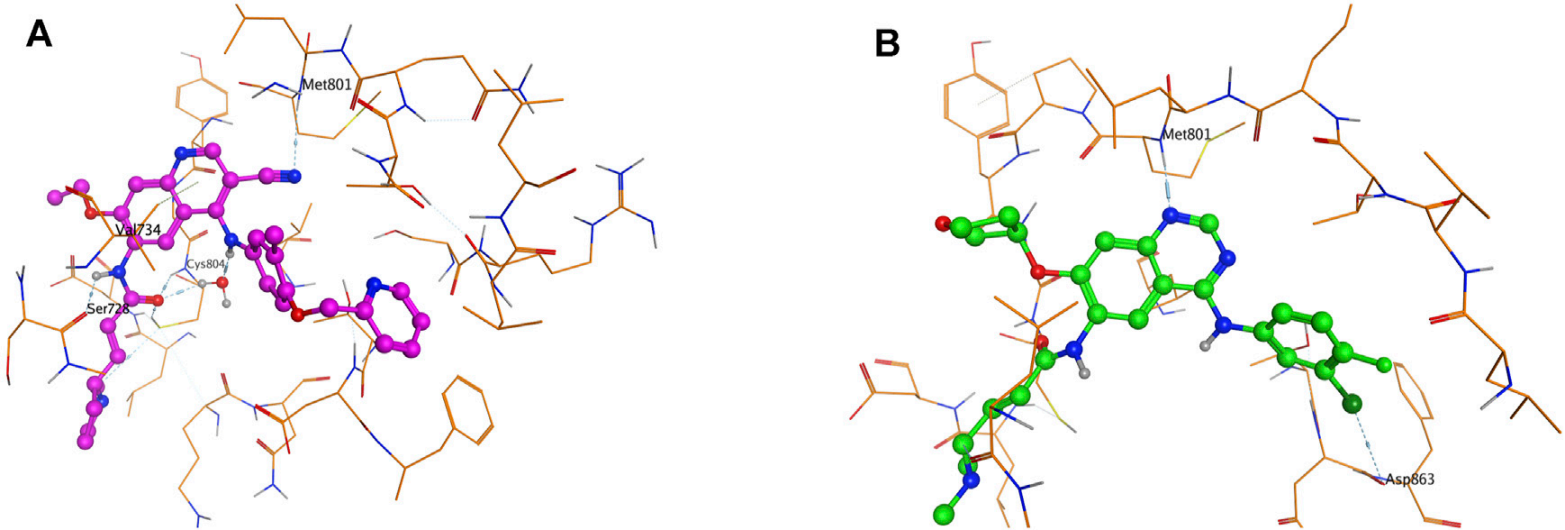
Drug binding modes of afatinib **(A)** and pyrotinib **(B)** for A775_G776insYVMA conformation.

Notably, the YVMA insertion causes a small segment of C-helix into the flexible loop region, with altering the *HER2* protein secondary structure greatly. The C-helix insertion is closer to the small molecules, which may form steric hindrance to TKI binding (Figure 3D). Molecular dynamics simulation issued that pyrotinib revealed superior binding activity to YVMA insertion than other inhibitors (Table 4). Afatinib revealed an inferior affinity to A775_G776insYVMA conformation by interaction of H-bond in Nitrogen-Met801 and Halogen bond in Chlorine-Asp863 by drug-binding simulation (Figure 4A). By comparison, pyrotinib potently bound A775_G776insYVMA conformation by interaction of more H-bonds in imino-Ser728, cyano-Met801, carbonyl-Cys804, imino and carbonyl-Water, and arene-H interaction in benzene-Val734 (Figure 4B).

**TABLE 4:** Binding energy of *HER2*-targeted TKIs towards *HER2* ex20ins variant A775_G776insYVMA.

Molecule	Δ*E*_*ele*_	Δ*E*_*vdw*_	ΔG_*bind*_ (MM/PBSA)	ΔG_*bind*_ (MM/GBSA)
Pyrotinib	−23.62	−73.87	−51.23	−57.91
Afatinib	−16.51	−53.25	−43.65	−46.42
Dacomitinib	−14.37	−51.38	−41.10	−41.73
Poziotinib	−4.61	−55.73	−37.41	−42.83

### Sensitivity to *HER2*-Targeted TKIs in YVMA Insertion and WT Cell Lines

**FIGURE 5:**
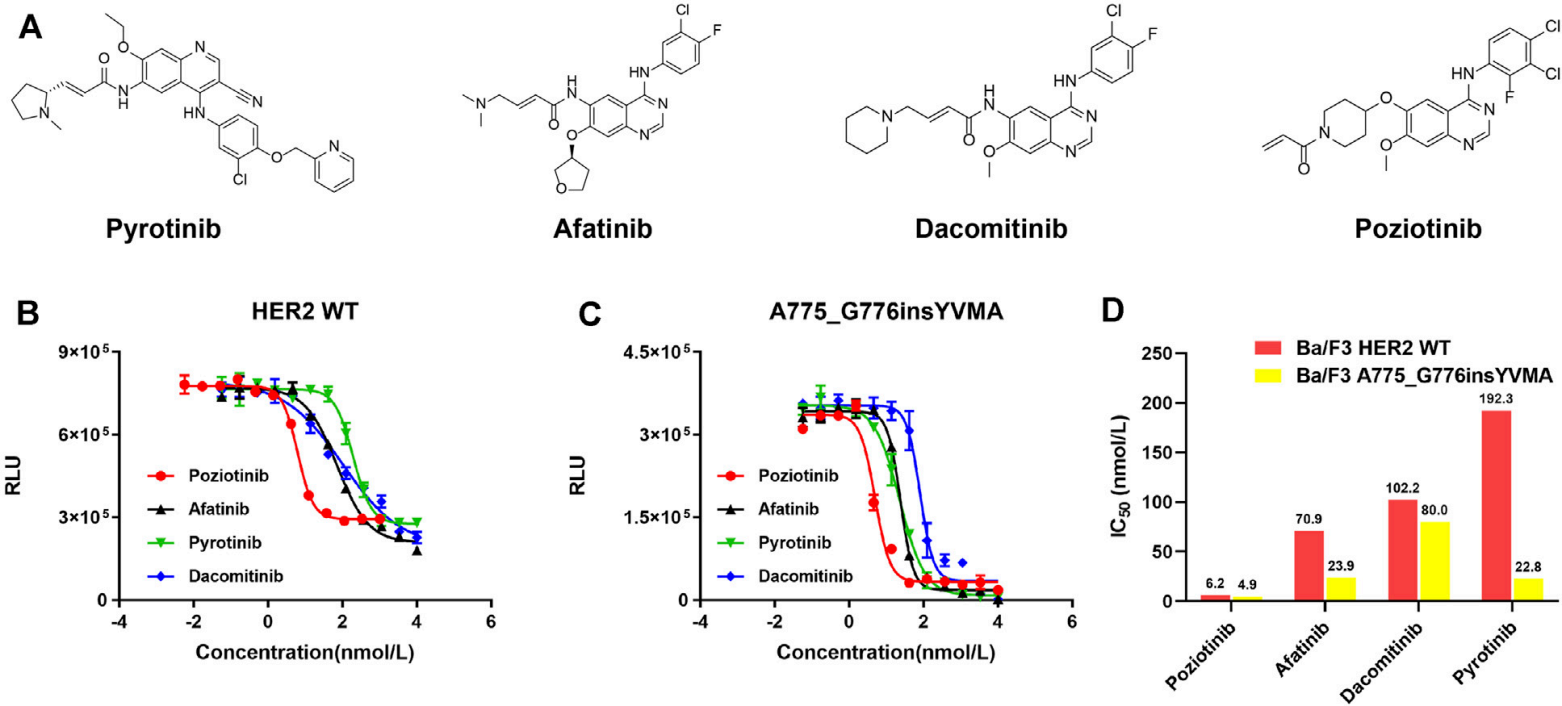
*HER2*-targeted TKIs pyrotinib, afatinib, dacomitinib, and poziotinib **(A)** were performed in cellular kinase inhibition assay to inhibit *HER2* wild type **(B)** and A775_G776insYVMA **(C)** Ba/F3 cells. IC_50_ values of above four inhibitors against *HER2* wild type and A775_G776insYVMA cells **(D)**.

For detecting cellular ATP, it is calculated as the total light emission amount-relative light unit (RLU). Cell viability assay and IC_50_ estimation were performed in *HER2* WT and YVMA Ba/F3 cells treated with four different inhibitors including pyrotinib, afatinib, dacomitinib, and poziotinib (Figure 5A). The RLU values correlated with the amount and viability of cultured cells, and poziotinib demonstrated very high potency against both *HER2* WT (Figure 5B) and YVMA cells (Figure 5C). Consistent with this finding, afatinib and dacomitinib revealed similarly potent inhibition against WT, but dacomitinib was less active against YVMA cells compared to afatinib. Pyrotinib showed the best selectivity to WT, with the least decreased RLU values, and it as well revealed more favorable activity to YVMA cells than afatinib and dacomitinib. The inhibition ability and IC_50_ values of four inhibitors against *HER2* WT and YVMA cells were depicted in Figure 5D.

## Discussion

To date, we present the first study to provide comprehensive evidence on differences of the binding activity between *HER2* exon 20 Gly776 deletion-insertions and A775_G776insYVMA to *HER2*-targeted TKIs afatinib and pyrotinib based on a real-world cohort investigation, along with structural insights into the mechanism by *in silico* analysis and cellular inhibition assay to give full exploration. We observed the specific *HER2* ex20ins subtype G776delinsVC with its similar variants G776delinsVV and G776delinsLC presented similar and favorable response to afatinib, but the predominant YVMA insertion showed poor response to it. In addition, pyrotinib revealed better efficacy and binding activity for these four insertion variants, with no obvious difference. We identified the specific *HER2* exon 20 Gly776 deletion-insertion variants could be as potential genomic modifiers of response to afatinib targeted therapy.

It has been reported that afatinib showed higher ORR (40%) and much longer mPFS (7.6 months) for ex20ins patients with G776delinsVC or G778_P780dup, compared with YVMA insertion (ORR, 0; mPFS of 1.2 months) (Fang et al., 2020). Among five patients with G776delinsVC, two achieved PR to afatinib, and the longest PFS (12.0 months) for afatinib was observed in one patient with G776delinsVC (Fang et al., 2020). Subsequent modeling of G776delinsVC revealed no obvious changes in the structure of drug-binding pocket over *HER2* WT. By comparison, structural modeling revealed YVMA conformation contained two bulky side chains, which may induce steric hindrance of the drug-binding pocket and thus preventing the interaction with afatinib (Fang et al., 2020). However, in the above study, the sample of patients with G776delinsVC was small (only five patients), and yet it had not analyzed the structural difference between G776delinsVC and its similar variants G776delinsVV and G776delinsLC, and it lacked molecular dynamics simulation for different *HER2*-targeted TKIs to provide evidence on the mechanism for binding activity differences.

Currently, there is not enough evidence to guide selective *HER2*-targeted TKIs for *HER2* ex20ins NSCLC patients. Consistent with our findings on afatinib, a series of studies had reported that afatinib showed limited activity for *HER2* ex20ins in NSCLC (De Grève et al., 2012; Liu et al., 2018; Nagano et al., 2018; Dziadziuszko et al., 2019; Lai et al., 2019; Fang et al., 2020). In addition, afatinib revealed much worse activity for the most common YVMA insertion compared with other insertion subtypes, with a limited ORR of 0–18.2% and mPFS of 1.2–3.0 months (Liu et al., 2018; Fang et al., 2020). By contrast, pyrotinib revealed mPFS of 6.9 months and overall ORR of 30% in patients with *HER2*-mutant advanced lung adenocarcinoma as second-line or above therapy, especially, with a higher ORR of 27.3% for YVMA insertion (Zhou et al., 2020).

In our study, afatinib showed mPFS of 2.9–3.4 months and ORR of 8.3–11.5% as 1L and 2L therapy for overall *HER2* ex20ins patients, and it revealed lower ORR of 0–9.5% and short mPFS of 2.8–3.2 months for YVMA insertion in 1L or 2L setting, which issued a much limited activity consistent with previous studies (Liu et al., 2018; Dziadziuszko et al., 2019; Fang et al., 2020). We also observed that G776delinsVC derived more survival benefit from afatinib than YVMA insertion (1L mPFS, 7.1 *vs.* 3.2 months; 2L mPFS, 4.3 *vs.* 2.8 months), in accordance with previous study reported (Fang et al., 2020). By comparison, pyrotinib showed PFS benefit than afatinib in overall patients no matter in 1L (median, 6.8 *vs.* 3.4 months) or 2L setting (median, 5.8 *vs.* 2.8 months). In addition, our previous PEARL study as well indicated that pyrotinib showed similar clinical benefit in patients with YVMA and non-YVMA insertions (mPFS, 5.1 *vs.* 5.2 months, *p* = 0.98), implying a broad spectrum of activity for distinct *HER2* ex20ins subtypes (Yang et al., 2021).

We subsequently constructed 3D models of these insertion variants, and observed no difference on drug binding pocket and TKI binding activity between G776delinsVC, G776delinsLC and G776delinsVV by structural analysis. Afatinib and pyrotinib showed favorable binding activities to these three deletion-insertions by molecular dynamics simulation. In our PEARL study, we comprehensively analyzed molecular differences between three *HER2* common ex20ins variants YVMA, G776delinsVC, P780_Y781insGSP, and concluded that pyrotinib showed better activity against the most common YVMA insertion, and with a similar activity to G776delinsVC compared with afatinib, dacomitinib, and poziotinib (Yang et al., 2021). Dynamics simulation in PEARL study also confirmed that ΔG_*bind*_ of pyrotinib was the lowest compared with other pan-*ErbB* inhibitors for YVMA and G776delinsVC insertions, owing to abundant H-bonds interaction with these insertions (Yang et al., 2021). The PEARL study did not compare G776delinsVC with its similar subtypes G776delinsVV and G776delinsLC, and we are uncertain if these insertions showed similar activities to *HER2*-targeted TKIs. Our current study makes a supplementary contribution to confirm that these three specific Gly776 deletion-insertions display favorable activity to afatinib and pyrotinib, especially, they are more sensitive to pyrotinib.

Of note, in this study, we found YVMA insertion significantly affected loop region following the C-helix with altering *HER2* protein secondary structure, thus forming steric hindrance to TKI binding, which was in accordance with our previous findings in PEARL study and other researches (Fang et al., 2020; Yang et al., 2021). As well, in this current study, we demonstrated that pyrotinib revealed superior binding activity to YVMA insertion by more and much potent H-bonds than afatinib, however, afatinib only interacted with YVMA insertion by H-bond in Nitrogen-Met801 and Halogen bond in Chlorine-Asp863 through 3D-based drug-binding simulation. Our PEARL study and this current research together confirmed that afatinib displayed inferior binding activity than pyrotinib for YVMA insertion, but they showed both favorable activities to *HER2* exon 20 Gly776 deletion-insertions. The cell viability assay and IC_50_ estimation results in this study finally supported our findings above, indicating that pyrotinib showed the best selectivity to *HER2* WT, and more potent antitumor activity to YVMA insertion than afatinib and dacomitinib. In our study, poziotinib demonstrated significantly potent antitumor activity in YVMA cells, which confirmed the reported findings in another study issuing that poziotinib was six times more potent than afatinib and dacomitinib in cell lines with *HER2* exon 20 mutants *in vitro* (Robichaux et al., 2018). Whereas, poziotinib as well exhibited high potency against *HER2* WT cells, which was correlated with high levels of *EGFR*-related toxicity, including rash and diarrhea requiring dose reductions in a significant number of patients (Elamin et al., 2021).

Though this real-world study precisely investigated the clinical activity of pyrotinib and afatinib for specific *HER2* ex20ins variants in advanced NSCLC, as well as providing valuable evidence from molecular dynamics simulation and cellular kinase inhibition experiment, several limitations must be noted. First, this was a retrospective real-world study that easily appeared selection bias. Moreover, the sample size of patients with *HER2* Gly776 deletion-insertions was small, which prevented us from providing further information on the clinical efficacy of *HER2*-targeted TKIs. Although we explained the potential mechanism on differences of binding activity to afatinib and pyrotinib in terms of specific insertion subtypes, this is an exploratory analysis based on structural modelling and molecular dynamics simulation that cannot fully represent all the possible reasons. More clinical evidence including Gly776 deletion-insertions cell lines and PDX models are warranted to correspond our findings, and so as to draw a clear conclusion.

In conclusion, both afatinib and pyrotinib showed favorable activity for NSCLC patients with *HER2* exon 20 Gly776 deletion-insertions. Pyrotinib revealed more potent activity to A775_G776insYVMA insertion than afatinib due to the steric binding hindrance induced by YVMA conformation.

## Data Availability

The original contributions presented in the study are included in the article/Supplementary Material, further inquiries can be directed to the corresponding author.

## Abbreviations

ATP, adenosine triphosphate; bp, base pair; CI, confidence interval; CR, complete response; DCR, disease control rate; DMSO, dimethyl sulfoxide; ex20ins, exon 20 insertion; HER2, epidermal growth factor receptor 2; IC_50_, half maximal inhibitory concentrations; MM/GBSA, molecular mechanics/Generalized Born solvent accessible surface area; MM/PBSA, molecular mechanics/Poisson-Boltzmann solvent accessible surface area; mPFS, median progression-free survival; NGS, next-generation sequencing; NSCLC, non-small cell lung cancer; ORR, objective response rate; PDX, patient-derived xenograft; PR, partial response; RECIST, Response Evaluation Criteria in Solid Tumors; RLU, relative light unit; SD, stable disease; TKI, tyrosine kinase inhibitor; WT, wild type; 1L, first-line; 2L, second-line; 3D, three-dimensional; ΔG_bind_, binding free energy; vs., versus; YVMA, A775_G776insYVMA.

## Appendix

The Δ*G*_*bind*_ of a protein-ligand complex is calculated in terms of the following relations.

$$\Delta G_{\mathrm{bind}}=\Delta G\left(\mathrm{complex}\right)-\left[\Delta G\left(\mathrm{protien}\right)+\Delta G\left(\mathrm{ligand}\right)\right]$$
(1)

$$\Delta G_{\mathrm{bind}}=\Delta E_{\mathrm{gas}}+\Delta G_{\mathrm{sol}}-T\Delta S$$
(2)

$$\Delta E_{\mathrm{gas}}=\Delta E_{\mathrm{ele}}+\Delta E_{\mathrm{ydw}}$$
(3)

$$\Delta G_{\mathrm{sol}}=\Delta G_{\mathrm{polar}}+\Delta G_{\mathrm{nonpolar}}$$
(4)

$$\Delta G_{\mathrm{nonpolar}}=\gamma \left(\mathrm{SASA}\right)+\beta$$
(5)

The gas-phase energies $\left(\Delta E_{\mathrm{gas}}\right)$ is composed of electrostatic energies $\left(\Delta E_{\mathrm{ele}}\right)$ and the van der Waals energy $\left(\Delta E_{\mathrm{ydw}}\right)$ . The solvation free energy $\left(\Delta G_{\mathrm{sol}}\right)$ is further decomposed into polar and nonpolar contribution. In addition, $\Delta G_{polar}$ is the polar solvation contribution calculated by solving the Poisson Boltzmann (PB) equations for MM/PBSA or generalized Born (GB) model for MM/GBSA method. $\Delta G_{nonpolar}$ is the nonpolar solvation contribution and is evaluated by the solvent accessible surface area (SASA) determined by a water probe radius of 1.4 Å. The corresponding solvating parameters *γ* and *β* are 0.00542 kcal/(mol Å) and 0.92 kcal/mol, respectively. T and S are the temperature and the total solute entropy, respectively. The change in conformational entropy—TΔS is usually calculated by normal-mode analysis on a set of conformational snapshots taken from MD simulations. In order to illustrate the interactions between inhibitor and each protein residue, the MM/GBSA decomposition analysis in Amber10 is performed.
